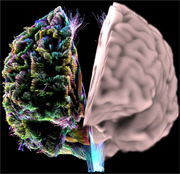# Dual Perspectives

**DOI:** 10.1002/alz.083822

**Published:** 2025-01-09

**Authors:** Tyler Ard, Ioannis Pappas

**Affiliations:** ^1^ Stevens Neuroimaging and Informatics Institute, University of Southern California, Los Angeles, CA USA; ^2^ Helen Wills Neuroscience Institute, University of California, Berkeley, CA USA

## Abstract

“Dual Perspectives” integrates multiple MRI scans, creating a nuanced synthesis of grey matter and diffusion‐based regional connections. This rendering holds particular significance in the realm of Alzheimer’s and dementia research by offering a comprehensive examination of data crucial for understanding these complex neurodegenerative conditions.

The inclusion of grey matter provides a detailed insight into the structural composition of the brain. Grey matter, comprised of neuronal cell bodies and dendrites, is fundamental to cognitive functions. High‐resolution grey matter imaging allows researchers to discern intricate structural alterations, contributing critical information about the anatomical changes associated with Alzheimer’s and dementia.

Simultaneously, the image delves into the realm of diffusion‐based regional connections, derived from the movement of water molecules within the brain’s white matter pathways. Given the pivotal role these pathways play in transmitting signals between different brain regions, understanding diffusion‐based connections becomes essential in unraveling the intricacies of Alzheimer’s and dementia.

The split harmonization of grey matter and diffusion‐based perspectives within this work offer a holistic view into the structural mediums of these neurodegenerative disorders. Overall, “Dual Perspectives” presents a lens into the intricacies of the brain, creating an artistic representation of neuroimaging‐derived data that is indispensable for advancing research in the field.